# Impact of Indocyanine Green Concentration, Exposure Time, and Degree of Dissolution in Creating Toxic Anterior Segment Syndrome: Evaluation in a Rabbit Model

**DOI:** 10.1155/2016/3827050

**Published:** 2016-07-12

**Authors:** Tamer Tandogan, Ramin Khoramnia, Gerd Uwe Auffarth, Michael Janusz Koss, Chul Young Choi

**Affiliations:** ^1^David J Apple International Laboratory for Ocular Pathology and International Vision Correction Research Centre (IVCRC), Department of Ophthalmology, University of Heidelberg, Im Neuenheimer Feld 400, 69120 Heidelberg, Germany; ^2^Department of Ophthalmology, Kangbuk Samsung Hospital, Sungkyunkwan University School of Medicine, 29 Saemunan-ro, Jongno-gu, Seoul 03181, Republic of Korea

## Abstract

*Purpose*. To investigate the role of indocyanine green (ICG) dye as a causative material of toxic anterior segment syndrome (TASS) in an experimental rabbit model.* Method*. Eight eyes of four rabbits were allocated to this study. Capsular staining was performed using ICG dye, after which the anterior chamber was irrigated with a balanced salt solution. The effects of different concentrations (control, 0.25, 0.5, and 1.0%), exposure times (10 and 60 seconds), and the degree of dissolution (differently vortexed) were investigated. The analysis involved anterior segment photography, ultrasound pachymetry, prostaglandin assay (PGE_2_ Parameter Assay, R&D systems, Inc.), and scanning electron microscopy of each iris.* Result*. There was no reaction in the control eye. A higher aqueous level of PGE_2_ and more severe inflammatory reaction were observed in cases of eyes with higher concentration, longer exposure time, and poorly dissolved dye. Additionally, scanning electron microscopy revealed larger and coarser ICG particles.* Conclusion*. TASS occurrence may be associated with the concentration, exposure time, and degree of dissolution of ICG dye during cataract surgery.

## 1. Introduction

Toxic anterior segment syndrome (TASS), an acute, noninfectious inflammation of the anterior segment of the eye, is a complication of anterior segment eye surgery, such as cataract extraction surgery. Various contaminants, usually from surgical equipment or supplies, have been implicated as causes of TASS [1]. Since 2000, there have been several reports of varying degrees of anterior segment damage from toxic substances after cataract surgery; some of these events had the appearance of an outbreak [2–6]. This led to the formation of the TASS task force, and the American Society of Cataract and Refractive Surgery (ASCRS) task force team reported 367 cases of TASS with multiple potential causative factors in 2010 [7].

TASS typically develops within 24 hours after surgery and is characterized by corneal edema and cellular reaction in the anterior chamber of the eye. Although most cases of TASS can be treated successfully with topical steroids, topical nonsteroidal anti-inflammatory agents, or both, the inflammatory response associated with TASS can cause serious damage to intraocular tissues, resulting in vision loss [8, 9].

**Figure 1:**
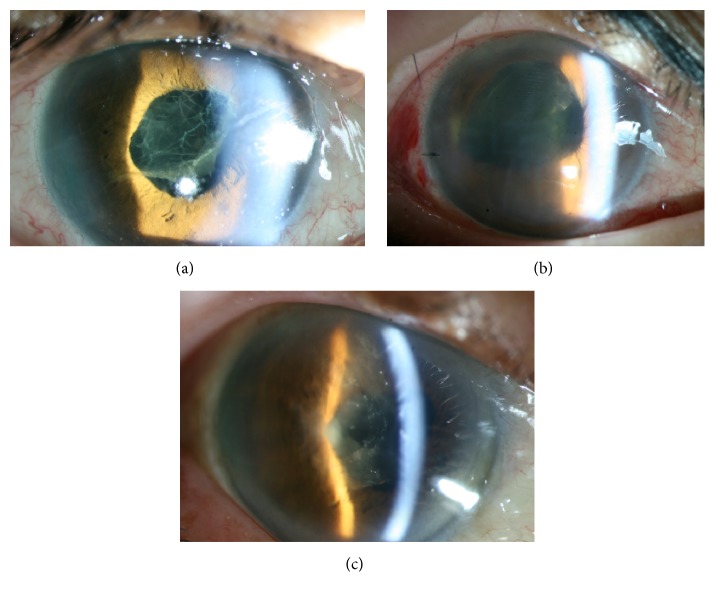
Corneal edema, severe inflammation, and fibrous membrane formation in the anterior chamber ((a) Patient 1, (b) Patient 2, and (c) Patient 3).

From October 2008 to November 2008, the Kangbuk Samsung Hospital Ophthalmology Surgical Center observed several patients who developed TASS after cataract surgery and who had been exposed to indocyanine green (ICG) dye for anterior capsular staining because of the presence of a mature senile cataract (Figure 1). The Ophthalmology Surgical Center had recently changed the dye for capsular staining from trypan blue to ICG. Trypan blue (Vision blue, DORC International, Zuidland, Netherlands) is commercially available and can be injected as it is, requiring no dilution. In contrast, dry ICG powder has to be mixed with diluents before administration. We suspected that ICG dye was the causative factor of the TASS cases described above. We therefore conducted this study to investigate whether ICG dye is a causative agent of intraocular inflammation in an experimental rabbit model.

## 2. Materials and Methods

This experimental study was approved by and conducted in compliance with IACUC protocol number 10-016-D1-N, 2010.12.16. It involved eight eyes of four male 2.5-month-old New Zealand white rabbits that weighed 2.5–3.0 kg. This was a preliminary study to evaluate the role of ICG with regard to postoperative inflammation, where we aimed to use as few rabbits as possible to evaluate the potential impact of ICG. Using the given study design and sample size, our aim was not to prove the effect statistically but rather to provide proof of principle.

ICG dye was used for anterior capsular staining in these rabbits, and the three physical variables of concentration, exposure time, and degree of dissolution were tested. There are several previous studies dealing with the safety and efficacy of ICG dye for capsular staining in cataract surgery [10, 11]. All animals were healthy and free of clinically detectable ocular disease. All the procedures involving animals were conducted in accordance with the Association for Research in Vision and Ophthalmology Resolution on the Use of Animals in Research. The experimental protocol was approved by the Institutional Animal Care and Use Committee of Kangbuk Samsung Hospital, Seoul, Korea. This work was supported by the Medical Research Funds from Kangbuk Samsung Hospital.

### 2.1. Preparation of Dye

The ICG dye was prepared by dissolving the commercially available product in aseptic distilled water (Diagnogreen, Daiichi Pharmaceutical, Tokyo, Japan). The control solution did not contain any ICG. According to previous studies [10, 11] the minimal optimum concentration for enhancement of anterior capsule visibility for capsulorhexis is 0.25% of ICG. Another experimental rabbit study confirms this concentration of 0.25% [12].

In this study, three concentrations of ICG (0.25%, 0.50%, and 1.00%) were prepared by dissolving ICG powder in 1.0 mL distilled water (provided with the ICG powder), and then these distilled-water solutions were diluted with 4.0 mL balanced salt solution (BSS, Alcon, Fort Worth, USA) to the final concentration of 0.5% ICG solution. To achieve concentrations of 0.25 and 1.0% ICG solutions, 1.0 mL distilled water with 9.0 mL BSS and 0.5 mL distilled water with 2.0 mL BSS were mixed, respectively, with 25 mg ICG granules. The calculated osmolarity values for each solution were 277.73, 250.45, and 250.5 mOsm (0.25%, 0.50%, and 1.0%), respectively. The pH measurements (Istek's Desktop pH meter, model 730P) of each solution at 37°C were 7.36 ± 0.05, 7.48 ± 0.05, and 7.44 ± 0.06 (0.25%, 0.5%, and 1.0%), respectively. To prepare these solutions, we used the approved clean bench with HEPA Filter and laminar flow system (Kastech, KT44-21C) in a facility dedicated solely to animal studies in Kangbuk Samsung Hospital Animal Study Laboratory. A vortex mixer (KMC-1300V, Vision Scientific, Seoul, Korea) was used to standardize dissolution, and the powder was dissolved for three minutes. This device allowed for more homogeneous dissemination of the granules and helped to avoid their accumulation in higher concentrations on different parts of the iris surface. This particular mixer device was utilized because it provided more consistent results than manual shaking, and it was a common device for mixing solutions.

### 2.2. Surgical Technique

General anesthesia was induced by intramuscular injection of zolazepam tiletamine (10 mg/kg; Zoletil, Virbac, Carros, France) and xylazine hydrochloride (0.5 mg/kg; Rompun, Bayer Healthcare, Seoul, Korea), supplemented with topical anesthetic (Alcaine, Alcon-Couvreur NV, Puurs, Belgium). A wire lid speculum was inserted to separate the eyelids. Two clear corneal incisions were made using a 1.0 mm diamond blade. One incision was used for the injection of the ICG solution and BSS for capsular staining or anterior chamber irrigation, respectively. The second incision served as the exit point for the irrigation solution. After a clear corneal incision was performed, air was injected into the anterior chamber to displace the aqueous humor. Then, with a 26-gauge cannula, 0.1 mL ICG solution was instilled onto the anterior capsule for the scheduled time, and the anterior chamber was washed with BSS. The time of exposure to ICG was 10 seconds or 60 seconds.

### 2.3. Description of Rabbits

 Rabbit Number One (ICG presence or absence) is described as follows:
   Right eye: 0.50% ICG dye prepared by vortexing for three minutes was administered for an exposure time of 60 seconds.
   Left eye (control): a mixture of distilled water and BSS without ICG was administered.
 Rabbit Number Two (difference in exposure time) is described as follows:
   Right eye: 0.50% ICG dye vortexed for three minutes was administered for 60 seconds.
   Left eye: 0.50% ICG dye vortexed for three minutes was administered for 10 seconds.
 Rabbit Number Three (difference in degree of dissolution) is described as follows:
   Right eye: 0.50% ICG dye vortexed for three minutes was administered for 60 seconds.
   Left eye: 0.50% ICG dye vortexed for 30 seconds was administered for 60 seconds.
 Rabbit Number Four (difference in ICG concentration) is described as follows:
   Right eye: 0.25% ICG dye vortexed for three minutes was administered for 60 seconds.
   Left eye: 1.00% ICG dye vortexed for three minutes was administered for 60 seconds.

### 2.4. Evaluation

Anterior segment photography, prostaglandin E_2_ (PGE_2_) competitive enzyme-linked immunosorbent assays (ELISA), and scanning electron microscopy were used to evaluate the eyes. Inflammatory reactions such as corneal edema, fibrin formation, and conjunctival injection were measured in the anterior segment with photography using slit lamp biomicroscopy (BX 900, Haag-Streit International, Koeniz, Switzerland) preoperatively and at 30 minutes, 12 hours, and 24 hours postoperatively. Within the cyclooxygenase cascade, PGE_2_ plays a major part in mediating the classical signs of inflammation and pain. Aqueous PGE_2_ levels can therefore serve as an indicator of the relative level of inflammation [13]. Twenty-four hours after the experiment, aqueous PGE_2_ concentrations were determined using a commercially available ELISA kit (PGE_2_ Parameter Assay, R&D Systems, Minneapolis, MN, USA). The minimum PGE_2_ quantification limit was 19.6 pg/mL, and the coefficient of variation was less than 11% within the calibration range (90–6000 pg/mL). Finally, eight rabbit iris specimens were excised for iris surface assessment using scanning electron microscopy (S-2380N, Hitachi, Tokyo, Japan).

## 3. Results

### 3.1. Anterior Segment Inflammation by Anterior Segment Photography

**Figure 2:**
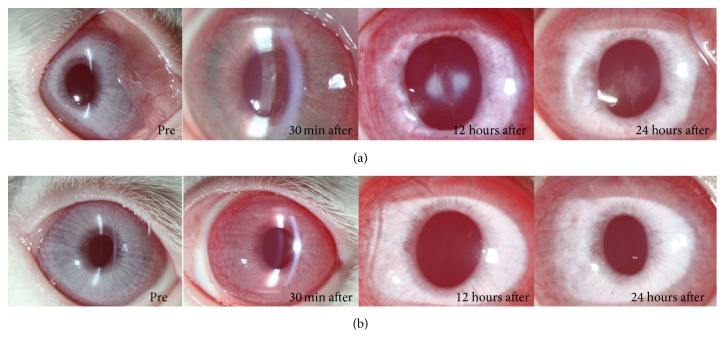
Fibrous membrane in the anterior chamber was observed immediately after applying ICG to the right eye of Rabbit Number One (a). It was more pronounced at 12 hours after the injection and then disappeared within 24 hours. The control eye was clear at all times (b).

**Figure 3:**
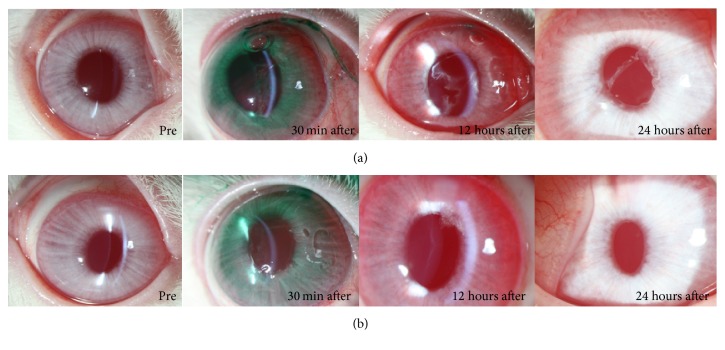
Compared to the ICG exposure time, fibrous membrane formation was more prominent in the right eye (a) of Rabbit Number Two that was exposed to ICG for a longer time than left eye (b).

**Figure 4:**
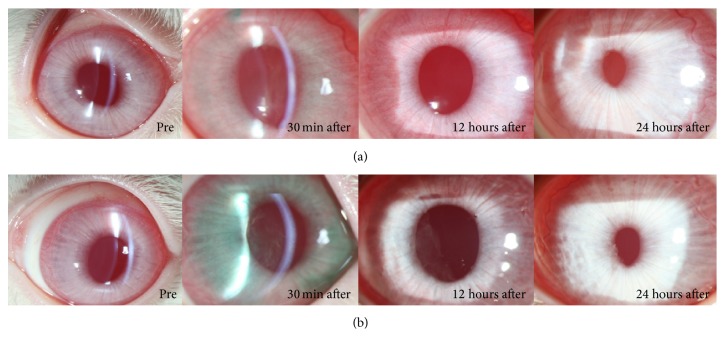
The right eye injected with ICG, dissolved for 3 minutes (a), and the left eye injected with ICG, dissolved for only 30 seconds (b). After 24 hours, the anterior chambers of both eyes were clear.

**Figure 5:**
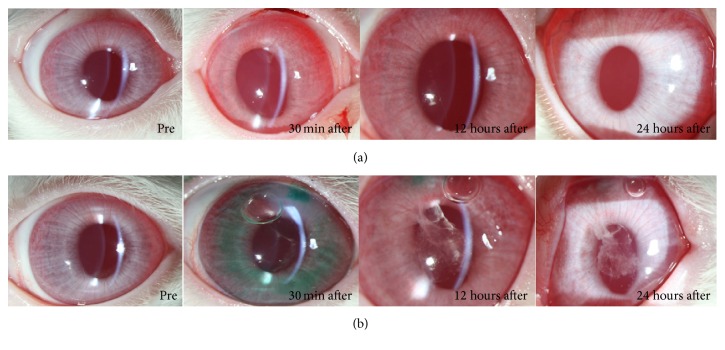
A very severe inflammation for up to 24 hours was observed in the eye to which 1.00% ICG had been applied (b). There was no inflammation in the eye treated with 0.25% ICG (a).

“Fibrous membrane” [14] formation in the anterior chamber was observed immediately after applying ICG to the right eye of Rabbit Number One. The fibrous membrane was more pronounced at 12 hours after the experiment and then disappeared within 24 hours (Figure 2(a)). The control eye (left eye) was clear at all times (Figure 2(b)). Fibrous membrane formation was more prominent in the right eye of Rabbit Number Two, which was exposed to ICG for a longer time than left eye with shorter exposure time (Figure 3). In the early period after the experiment, the left eye, into which ICG had been injected, which was dissolved for only a short period of time (30 seconds), was more inflamed than the right eye, which was treated with a more dissolved solution of ICG. However, after 24 hours, the anterior chambers of both eyes were clear (Figure 4). A very severe inflammatory reaction for up to 24 hours was observed in the eye to which 1.00% ICG had been applied. There were no inflammatory reactions in the eye treated with 0.25% ICG (Figure 5).

### 3.2. Quantification of Aqueous Prostaglandin E_2_ Level

**Figure 6:**
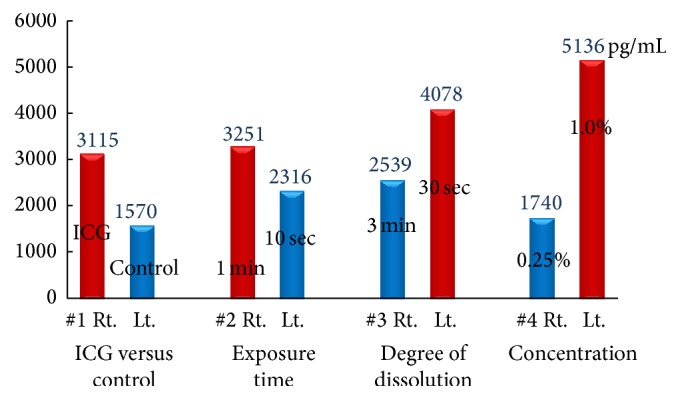
The PGE_2_ concentrations in aqueous humor. The rabbit eyes exposed to ICG for a longer period of time (60 seconds), ICG dissolved for only a short time (30 seconds), or a high concentration of ICG (1.00%) resulted in high PGE_2_ levels.

The results of prostaglandin E_2_ ELISA analysis correlated well with those of anterior segment photography. The rabbit eyes exposed to ICG for a longer period of time (60 seconds), ICG dissolved for only a short time (30 seconds), or ICG with a high concentration (1.00%) resulted in high PGE_2_ levels. The most significant difference was observed between eyes treated with different concentrations of ICG (Figure 6).

### 3.3. Assessment of Residual ICG Dye by Scanning Electron Microscopy

**Figure 7:**
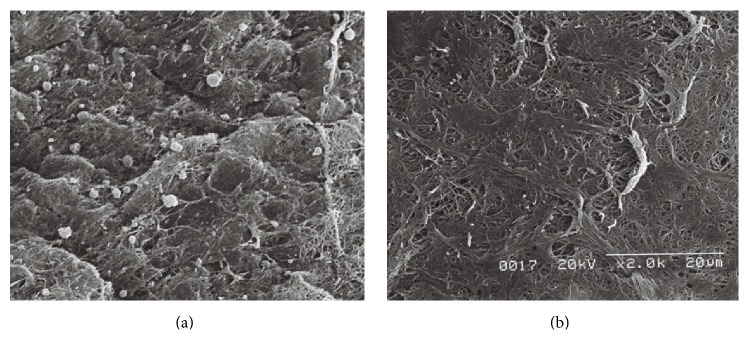
Residual ICG particles on the iris surface were observed using scanning electron microscopy. There were many ICG granules on the iris of the right eye of Rabbit Number One, ranging in size from 1 to 5 *μ*m (a). However, no similar granules were observed in the control eye (b).

**Figure 8:**
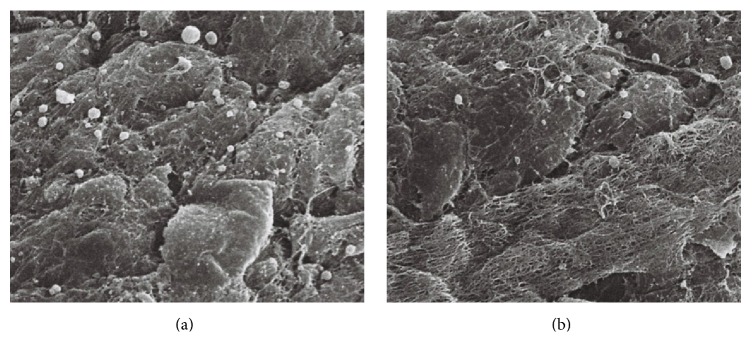
The right eye of Rabbit Number Two, which was exposed to ICG for 60 seconds, had slightly more granules (a) than the left eye (b).

**Figure 9:**
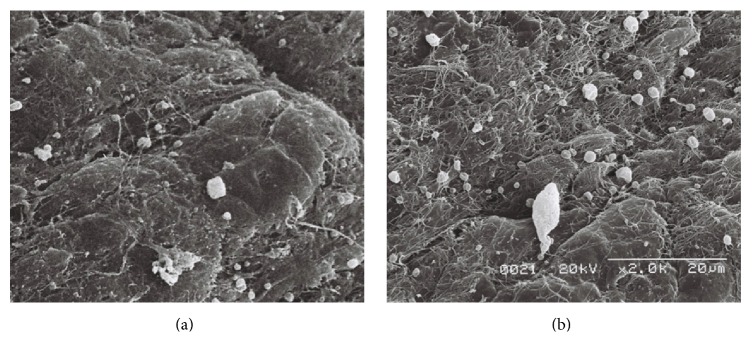
The left eye of Rabbit Number Three treated with ICG dissolved for only 30 seconds had larger white granules (b) compared with the right eye, which was treated with ICG, that was vortexed for a longer time (a).

**Figure 10:**
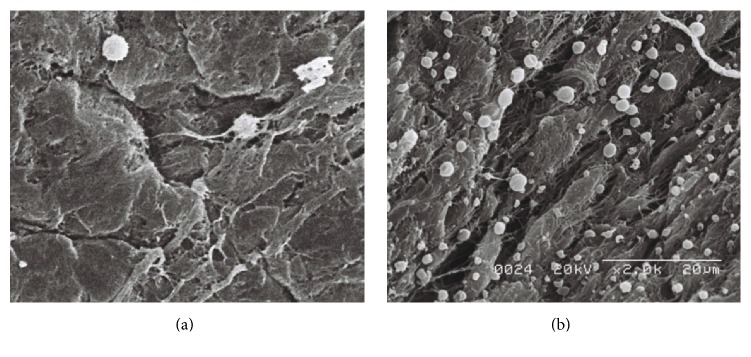
There were numerous granules in the left eye of Rabbit Number Four, which was treated with 1.00% ICG (b). In contrast, very few remnant ICG particles were observed in the right 0.25% ICG-treated eye of this rabbit (a).

Residual ICG particles on the iris surface were observed using scanning electron microscopy. ICG granules were identified based on their presence and morphology compared to the control image. As there could not physiologically be electron dense round particles on the iris, these particles were characterized as ICG granules. Furthermore, the ICG granules were also completely different in size and shape when compared with inflammatory cells, fibers, and muscular structures. There were many ICG granules on the iris of the right eye of Rabbit Number One, ranging in size from 1 to 5 *μ*m. No similar granules were observed in the control eye (Figure 7). The right eye of Rabbit Number Two, which was exposed to ICG for 60 seconds, had slightly more granules than the left eye (Figure 8). The left eye of Rabbit Number Three treated with ICG dissolved for only 30 seconds had larger white granules compared to those in the right eye of the same rabbit treated with ICG dissolved for three minutes (Figure 9). The large sizes of these granules on the left eye may be related to incomplete dissolution caused by shorter mixing time. There were numerous granules in the left eye of Rabbit Number Four, which was treated with 1.00% ICG. In contrast, very few remnant ICG particles were observed in the right eye of this rabbit, which was treated with 0.25% ICG (Figure 10).

## 4. Discussion

There are several factors influencing anterior chamber inflammation during cataract surgery, such as insufficient mydriasis, elongated surgery time, excessive use of ultrasonic energy during phacoemulsification, iris incarceration into the corneal incisions, iris damage through phaconeedle, and irritating or toxic foreign materials. In this study we isolated and analyzed the role of ICG as a factor for inflammation in the anterior chamber.

Our results based on an animal model suggest that residual intraocular indocyanine green (ICG) dye can cause postoperative inflammation in the anterior segment. In this preliminary study to evaluate the role of ICG with regard to postoperative inflammation, the study design and sample size were optimized to harm as few rabbits as possible in order to provide proof of principle rather than prove a statistical effect.

Although toxic anterior segment syndrome was first described by Monson et al. [15] in 1992, its exact pathophysiology still remains unclear. It is known, however, that toxic foreign substances that intra- or postoperatively enter the anterior chamber can cause inflammation.

The common causes of TASS, as described by the ASCRS task force team, include improper cleansing of surgical equipment, use of enzymatic cleaners, an inappropriate detergent concentration, and antibiotics or preservatives for antibiotics that are intraoperatively mixed with an irrigation solution [7]. However, no prior study has reported yet that ICG dye used for anterior capsular staining can cause TASS; trypan blue and ICG are both used as a capsular staining agent [16–21].

Trypan blue is commercially available in liquid form, whereas ICG comes in powder form and needs to be dissolved in a solvent before use. In countries such as South Korea where there are legal issues surrounding the intraocular use of trypan blue and/or import difficulties, ICG is used for anterior capsular staining. Generally, a 0.5% ICG solution is used for anterior capsular staining [10]. No standardized guidelines on methods of preparation or use of the solution are available. As such, there is a possibility that the concentration of the prepared ICG solution could be higher than required or that the dye may not be properly dissolved. In addition, if the anterior chamber is exposed to the dye solution for too long after injection of the solution or if the anterior chamber, especially the iris, is insufficiently washed, residual ICG particles may cause inflammation. We therefore performed* in vivo* experiments in rabbits to determine how the factors related to administration of ICG such as time of exposure to ICG, degree of dissolution, and concentration may influence the intraocular inflammatory reaction caused by ICG.

In this study, anterior chamber inflammation was found to be severe, and scanning electron microscopy of the iris surface showed more residual ICG particles when the time of exposure was long (60 seconds) and when the dissolving time was short (30 seconds). The degree of inflammation was much more severe and there were more residual ICG particles on the iris surface when 1.00% ICG was used compared to those when 0.25% ICG was used. The concentration of PGE_2_ in the aqueous humor was measured to quantify the degree of anterior chamber inflammation. We found that the higher the degree of anterior chamber inflammation was, the higher the level of PGE_2_ was in the aqueous humor.

Based on these findings, we hypothesize that insoluble particles that remain in the ICG solution attached to the iris surface or to the anterior chamber angle during anterior capsular staining without being completely washed off can cause inflammation. Thus, if the formation of insoluble ICG particles can be minimized, inflammation caused by residual ICG particles could be reduced. The dissolution time, solution concentration, and solvent type all contribute to the formation of insoluble ICG particles. Generally, a mixed solution of distilled water and BSS is used to prepare ICG dye solution. Nishimura et al. [22] reported that insoluble ICG particles could form when BSS was used to prepare the ICG dye solution.

A preliminary experiment was performed using different ICG concentrations and different ratios of the two solvents [distilled water and BSS], and we observed a difference in the amounts of insoluble particles that were formed using a light microscope. We found that the higher the ICG concentration and the proportion of BSS in the solvent, the greater the proportion of insoluble particles (data not shown). This is likely to be related to the saturability of the solution. Of the substances used, the one which is most similar to aqueous humor in composition is BSS. As BSS contains various ions and molecules, its upper limit of complete dissolution of ICG is 0.5%. If the ratio of distilled water is increased to achieve complete dissolution of ICG, however the pH and osmolality of the ICG dye solution could decrease, which would adversely affect the anterior segment structures. Thus, sufficient time is required to dissolve ICG in a mixed solution containing a higher ratio of BSS to distilled water in order to maintain the regular intraocular environment.

Parikh and Edelhauser [23] experimentally showed that corneal edema with TASS is likely due to direct corneal endothelial toxicity. However, in this study, the postoperative complications related to the iris were more pronounced than corneal decompensation. The concentration and saturability of ICG presumably all play a role in the anterior chamber inflammation. The mesh-like characteristic of the iris structure may function as a reservoir for the remaining particles. Therefore, the postoperative inflammation is more likely related to the iris than to the cornea. Thus, ICG-related anterior chamber inflammation, such as fibrin formation, could occur.

The limitations of this study are as follows: we only used a small number of rabbits; the pupil was undilated when anterior capsular staining was performed, which increased the possibility of ICG particles remaining on the iris surface; the amount of insoluble ICG in the anterior chamber could not be quantitatively measured according to exposure time, dissolution time, or concentration. There might also be differences in the inflammatory reactions of humans and rabbits. The difference in osmolarity values could also affect the inflammatory reactions after exposure of ICG solution in the anterior chamber. Nevertheless, we demonstrated for the first time that ICG can cause intraocular inflammation. In addition, we showed that the concentration of ICG, time of exposure to ICG, and the degree of dissolution of ICG affected the severity of inflammation. Anterior segment photography, scanning electron microscopy, and aqueous PGE_2_ ELISA objectively showed that the greater the number of residual intraocular ICG particles was, the greater the severity of inflammation was.

These results show that, in the absence of trypan blue, ICG could also be used with caution as a capsular dye. In this case, special care should be taken to ensure a proper dissolution rate, lower concentration, and thorough cleansing of the ICG from the anterior chamber after staining the anterior capsule of the lens to avoid ICG granules from being captured within the meshwork-like structures of the iris surface which can otherwise cause significant anterior chamber inflammation. Chang et al. [12] suggest that the minimal concentration of ICG dye to show similar visibility with ICG 0.5% is ICG 0.25%. We also suggest a similar conclusion that the lower concentration of ICG dye can be a safer option for cataract surgery in terms of saturability, pH, and osmolarity of ICG.

Further studies are required to investigate other substances that can be related to the occurrence of TASS.
